# The Role of the Human Brain Neuron–Glia–Synapse Composition in Forming Resting-State Functional Connectivity Networks

**DOI:** 10.3390/brainsci11121565

**Published:** 2021-11-27

**Authors:** Sayan Kahali, Marcus E. Raichle, Dmitriy A. Yablonskiy

**Affiliations:** 1 Department of Radiology, Washington University in Saint Louis, Saint Louis, MO 63110, USA; kahalisayan@wustl.edu (S.K.); mraichle@wustl.edu (M.E.R.); 2 Department of Neurology, Washington University in Saint Louis, Saint Louis, MO 63110, USA

**Keywords:** functional connectivity networks, brain cellular composition, quantitative Gradient-Recalled Echo, qGRE, MRI, default mode network

## Abstract

While significant progress has been achieved in studying resting-state functional networks in a healthy human brain and in a wide range of clinical conditions, many questions related to their relationship to the brain’s cellular constituents remain. Here, we use quantitative Gradient-Recalled Echo (qGRE) MRI for mapping the human brain cellular composition and BOLD (blood–oxygen level-dependent) MRI to explore how the brain cellular constituents relate to resting-state functional networks. Results show that the BOLD signal-defined synchrony of connections between cellular circuits in network-defined individual functional units is mainly associated with the regional neuronal density, while the between-functional units’ connectivity strength is also influenced by the glia and synaptic components of brain tissue cellular constituents. These mechanisms lead to a rather broad distribution of resting-state functional network properties. Visual networks with the highest neuronal density (but lowest density of glial cells and synapses) exhibit the strongest coherence of the BOLD signal as well as the strongest intra-network connectivity. The Default Mode Network (DMN) is positioned near the opposite part of the spectrum with relatively low coherence of the BOLD signal but with a remarkably balanced cellular contents, enabling DMN to have a prominent role in the overall organization of the brain and hierarchy of functional networks.

## 1. Introduction

Resting-state functional brain connectivity has become an established area of research in the arena of cognitive neuroscience and its related applications. Functional connectivity refers to the statistical correlation between temporally coherent low-frequency spontaneous fluctuations of the resting-state functional MRI (rs-fMRI) signal in different brain regions [1] and provides insight into the large-scale brain circuit organization [2,3]. The rs-fMRI signal is acquired with MRI sequences sensitive to the Blood–Oxygen Level-Dependent (BOLD) effect [4] and identifies consistent resting-state networks [5,6,7] that play important roles in both normal brain function and various neurological conditions, such as, for example, normal aging [8] and Alzheimer disease [9].

An important question in understanding the physiological basis of resting-state functional connectivity is its relationship to brain structural connectivity and brain cellular composition. In the human brain, the structural connectivity issue is usually addressed by studying trajectories of brain white matter (WM) tracts with Diffusion Tensor Imaging (DTI) tractography [10,11,12,13]. However, the direct structural connectivity through WM is not the sole mechanism underlying functional connectivity [14,15].

While DTI is sensitive to structural connections governed by WM tracts, it does not usually have enough sensitivity to resolve existing neuronal connections through the brain cortical regions, which are characterized by a complex network of interconnected and intersected neuronal processes (axons and dendrites). It is also understood that the functional connectivity of the brain is not uniquely dependent on the neurons; rather, it also relies on glial cells which significantly influence structural and functional connectivity [16], providing metabolic and regulatory support for neurons [17,18,19]. Astrocytes are responsible for increasing the amount of mature and functional synapses [17,18]. Moreover, neuron–glia cross-talk leads to synaptic formation and remodeling [20]. It has also been observed that neurons generate weak synapses in the absence of glia [17,21]. Hence, incorporating information on cellular composition into the brain structural connectivity can provide crucial information for understanding the brain functional connectivity network formation and functioning.

**Figure A1:**
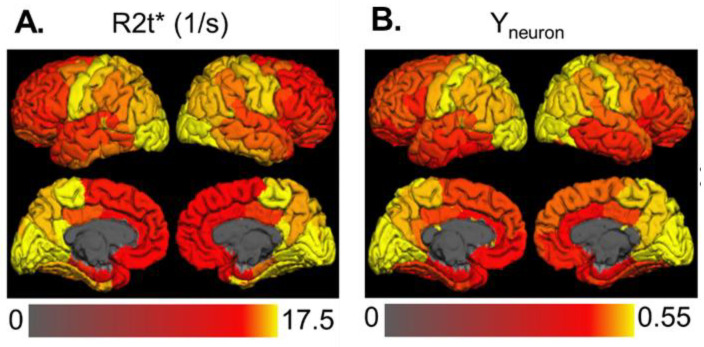
The association between the expression of neuron-related genetic networks across different regions of the human brain and R2t* measurements in the same regions (adapted from [26]). The R2t* map (**A**) in this figure was obtained from qGRE MRI; Yneuron (**B**) designated as the Neuronal Density Index) was obtained from the analysis of the gene expression profiles in the Allen Human Brain Atlas, including two totally independent measurements. The figure and analysis in [26] provided convincing and consistent with literature arguments that the R2t* metric of the qGRE signal is strongly associated with neuronal density.

In this paper, to identify brain cellular structure, we use data obtained by means of the quantitative Gradient-Recalled Echo (qGRE) MRI technique [22]. The qGRE method is based on the gradient-recalled echo MRI with multiple gradient echoes and data analysis, allowing for the separation of tissue cellular-specific (R2t*) GRE signal relaxation from relaxation caused by the *baseline* BOLD mechanism [4,23] and by adverse effects of magnetic field inhomogeneities [24] corrected using the VSF (voxel spread function) method [25]. We also use a quantitative relationship established in Wen et al. [26] between the brain cellular composition and the R2t* metric of the qGRE MRI signal. Based on the analysis of the genetic information from the Allen Human Brain Atlas, Wen et al. [26] identified several networks of gene expression profiles coherently expressed across brain anatomical structures and established their association with the R2t* metric of the qGRE signal in these structures. Data showed the strongest association between R2t* and genes that are associated with ion channels primarily distributed along neuronal processes, including axons (myelinated and non-myelinated) and dendrites, which typically occupy over 85% of the space comprised by neurons. In noting these findings, it was demonstrated [26] that the in vivo R2t* measurement largely reflects a transcriptional correlate for major parts of the neuronal cell bodies and processes, thus representing the neuronal contribution to brain tissue cellular composition. Moreover, a quantitative relationship between the R2t* metric of the qGRE signal in healthy human brain cortical Gray Matter (GM) and the Neuronal Density Index *Y_neuron_* that serves as a proxy for brain tissue neuronal density was established in [26]:

(1) $$R2t^{*}=5.8+20.4\cdot Y_{neuron}$$

where R2t* is measured in sec^−1^ and the Neuronal Density Index *Y_neuron_* is a dimensionless parameter (0 < *Y_neuron_* < 1) proportional to the tissue neuronal density. This relationship is illustrated in Appendix A, Figure A1. Further analysis [26] also established relationships in healthy human brain cortical GM between *Y_neuron_* and indices characterizing densities of both glia cells (*Y_glia_*) and synapses (*Y_synapse_*):

(2) $$Y_{glia}/Y_{neuron}=0.38\cdot Y_{neuron}^{-1.38};\;Y_{synapse}/Y_{neuron}=0.34\cdot Y_{neuron}^{-1.51}$$

Thus, these findings corroborated previous histology-based reports [27,28] that healthy human brain regions with higher neuronal density have relatively low densities of glia cells and synapses.

To further understand the role that different parts of the neuron might play in the formation of brain functional connectivity, we compare our R2t*-based results with a ratio of T1-weighted (T1w) to T2-weighted (T2w) images as a proxy related to cortical tissue myelin content as proposed by Glasser and Van Essen [29].

For rs-fMRI, we provide data analysis based on a structure of 300 parcels (functional units) combined in 17 resting-state networks developed by Yeo et al. [7,30]. We have selected this brain parcellation scheme [30] because it reveals neurobiologically meaningful features of brain organization, providing a sufficient number of parcels to delineate brain cortical anatomical structures, thus satisfying important features discussed by Van Essen et al. [31]. Since fMRI signals in each functional unit exhibit high levels of homogeneity [7,30], we characterize fMRI signals inside the functional units by their average (across voxels/vertices comprising these units) values and then use these average signals to calculate the resting-state functional connectivity strength between functional units for each network.

Since only the infra-slow (below 0.1 Hz) frequency fluctuations of the resting-state BOLD signal are usually considered as a signature of neuronal activity [3,32,33,34,35,36], we also investigate the detailed relationship between the cellular composition and frequency components of the resting-state BOLD signal.

In this paper, by combining qGRE-derived brain cellular information with resting-state BOLD data from the Human Connectome Project (HCP), we establish a quantitative relationship between resting-state functional connectivity and human brain cortical cellular composition. We show that while the synchrony of the brain cellular circuits in network-defined functional units is mainly associated with the regional neuronal density, the strength of the inter-unit within-network functional connectivity has significant association not only with units’ neuronal content but also with their glia and synaptic constituents. These cellular-functional associations are most prominent in the infra-slow frequency range (0.01–0.16 Hz) of brain activity as determined by the BOLD signal.

## 2. Materials and Methods

### 2.1. Resting-State Functional Connectivity Data

The functional connectivity data were obtained from the HCP1200 dataset (February, 2017 release). The HCP1200 dataset was recorded on young adults aged between 22 and 35 years [5]. In this study, we used 183 subjects (about 20% of the total available) that had four 15-min RS-fMRI scans. The fMRI scans were preprocessed using HCP structural (PreFreeSurfer, FreeSurfer, and PostFreeSurfer) and functional (fMRIVolume and fMRISurface) pipelines. The ICA+FIX pipeline was applied to refine the rs-fMRI data to regress out the spatially specific temporal artefacts, i.e., scanner artefacts, subject movement, breathing, and cardiac pulsation. The fMRI data were then summarized into 91,282 grayordinates, which are cortical gray matter surface vertices and subcortical gray matter voxels [37]. The data in each grayordinate obtained from the four scans of each subject (1200 timepoints × 4 runs) were concatenated after performing de-meaning and variance normalization. The concatenated time series data for each subject were grouped into 300 ROIs (i.e., functional units) using the local–global parcellation of the cortical gray matter of Schaefer et al. [30], identified by employing the gradient-weighted Markov Random Field (gwMRF) model which fuses local gradient and global similarity approaches. Signals from these time-series data for all vertices in each ROI were averaged together and each ROI for further analysis was represented by a single signal (1200 timepoints × 4 runs). These time-series data for each of the 183 subjects were concatenated together for time-domain network analysis (1200 timepoints × 4 runs × 183 subjects).

To quantitatively characterize rs-fMRI signals (i.e., the statistical correlation between temporally coherent spontaneous fluctuations of the rs-fMRI signal in different brain regions), we introduced a short-range signal coherence (signal coherence in individual functional units) and a long-range signal coherence (signal coherence between functional units).

To represent the signal coherence of the resting-state functional BOLD signal from each individual functional unit (designated below as region of interest, i.e., ROI), we calculated the standard deviation (STD*_i_*) of the average signals combined from individual vertices in each functional unit *i* (*i* *=* 1, 2,…, 300). Due to normalization, the STD of each signal from the vertices is equal to one, such that the STD*_i_* of the combined signal represents *the coherence* of the individual signals in the ROI (STD = 1 if signals from vertices are coherent and STD = 0 if there is no correlation between them). Then, we represented the BOLD signal coherence for each network as a mean value of STD*_i_* of all ROI*_i_* belonging to a given network:

(3) $$\mathrm{BOLD}\;\mathrm{Signal}\;{\mathrm{Coherence}}_{n}=\mathrm{mean}\langle {\mathrm{STD}}_{i}\rangle ;\;n=1,2,\ldots ,17$$

The resting-state functional connectivity strength (CS*_i_*_,*j*_) between two ROIs (*i* and *j*), i.e., the signal coherence between functional units, is calculated as a Pearson Correlation Coefficient between two time series data from ROI*_i_* and ROI*_j_*, each having 1200 timepoints × 4 runs × 183 subjects.

To characterize the intra-network connectivity, we introduced the intra-network connectivity strength NCS*_n_* by calculating the weighted average CS*_i_*_,*j*_ between all ROIs belonging to a given network:

(4) $${\mathrm{NCS}}_{n}=\mathrm{mean}{\langle {\mathrm{CS}}_{i,j}\rangle }_{i,j};\;n=1,2,\ldots ,17$$

Since ROIs contain different number of vertices, all ROIs in this calculation were weighted by the number of the corresponding vertices.

The frequency content of the rs-fMRI signal was analyzed by converting the time-series data to the frequency domain by performing a Fourier transformation on time series data from each ROI independently for each session per subject. The Fourier transformed data were then averaged across all sessions and all subjects before separation into preselected frequency bins with the widths of 0.014 Hz and 0.0047 Hz. Signals from each bin were then converted back to the time domain using an inverse Fourier transform.

### 2.2. Quantitative Gradient-Recalled Echo (qGRE) MRI Data Analysis

To identify the brain cellular structure, we used data obtained by means of the quantitative gradient-recalled echo (qGRE) MRI technique [22]. The qGRE method is based on the gradient-recalled echo MRI with multiple gradient echoes and data analyses, allowing for the separation of tissue cellular-specific (R2t*) GRE signal relaxation from the relaxation caused by the baseline blood–oxygen level-dependent (BOLD) mechanism and by adverse effects of magnetic field inhomogeneities [24]. We used MRI data obtained from 16 healthy volunteers aged between 23 and 35 years from a previously published study [38]. These volunteers were not part of the HCP sample but their age range was selected to match HCP resting-state functional MRI data. In Zhao et al. [38], MRI image data were obtained using a 3T Trio MRI scanner (Siemens, Erlangen, Germany) using a 32-channel phased-array RF head coil. Data acquisition was done by means of a 3D gradient-recalled echo (GRE) MRI sequence with 10 gradient echoes, followed by a navigator for correcting physiological fluctuations [39]. The sequence parameters were flip angle (FA) = 30°, repetition time (TR) = 50 ms, first echo time (*TE*_1_) = 4 ms, echo spacing Δ*TE* = 4 ms, voxel size of 1 × 1 × 2 mm^3^, and an acquisition time of 11 min. Field inhomogeneity effects were corrected using the Voxel Spread Function (VSF) method [25].

(5) $$S_{n}\left(TE\right)=\sum_{ch=1}^{M}\lambda_{ch}\cdot {\bar{S}}_{n}^{ch}\left(TE_{1}\right)\cdot S_{n}^{ch}\left(TE\right),\;\lambda_{ch}=\frac{1}{M\cdot \varepsilon_{ch}^{2}}\sum_{ch^{\prime }=1}^{M}\varepsilon_{ch^{\prime }}^{2}$$

where $\bar{S}$ denotes the complex conjugate of *S*; index *n* represents the voxel position in space; *λ_ch_* are weighting factors; and *ε_ch_* are noise amplitudes (r.m.s.).

A theoretical model of the BOLD (blood–oxygen level-dependent) contrast [23] was used to differentiate the contribution of tissue cellular-specific relaxation (R2t*) and BOLD contributions to the total R2* relaxation:

(6) $$S(TE_{n})=A_{0}\cdot \exp\left[-R2t_{}^{*}\cdot TE_{n}-\zeta \cdot f_{s}(\delta \omega \cdot TE_{n})-i\cdot \varphi -i\cdot 2\pi \cdot \Delta f\cdot TE_{n}\right]\cdot F(TE_{n})$$

where *A*_0_ is the signal amplitude; *δω* is the characteristic frequency determined by the susceptibility difference between deoxygenated blood and the surrounding tissue; ζ is the volume fraction of deoxygenated blood; the non-linear function $f_{s}\left(\delta \omega \cdot TE\right)$ accounts for the BOLD effect [23]; *φ* and Δ*f* are phase and frequency shifts (dependent on tissue structure and also on the macroscopic magnetic field created mostly by tissue/air interfaces); and the function *F*(*TE*_n_) describes the effect of macroscopic magnetic field inhomogeneities [24]. Here, *F*(*TE*_n_) was calculated by the voxel spread function (VSF) method [25] and we used a mathematical expression for the function fs in terms of a generalized hypergeometric function [40] F21:

(7) fs(δω⋅TE)=F21([−12];[34,54];−916(δω⋅TE)2)−1

### 2.3. Structural Connectivity Analysis Based on qGRE-Defined Brain Cellular Structure

For our analysis, the R2t*-based calculated cellular indices for each subject, expressed in Equations (1) and (2), were projected on the cortical surface through the surface-based registration using the grayordinates. The grayordinates were then combined into 300 ROIs based on the parcellation of Schaefer et al. [30]. After that, the results for all 16 subjects were averaged for each of the ROIs, producing 300 sets of cellular indices (*Y_neuron_*_,_ *Y_glia_*, and *Y_synapse_*).

To establish a joint contribution of two distinct ROIs (*i* and *j*) to their resting-state functional connectivity strength *CS_i,j_*, we considered six cellular association (*CA_i,j_*) matrices depending on their cellular composition, such as neuron–neuron, glia–glia, synapse–synapse, neuron–glia, neuron–synapse, and glia–synapse, which can be defined on 300 ROIs as follows:

(8) $$\begin{array}{l} CA_{neuron-neuron}=\langle Y_{neuron}^{i}\times Y_{neuron}^{j}\rangle ;\;CA_{glia-glia}=\langle Y_{glia}^{i}\times Y_{glia}^{j}\rangle ;\;CA_{synapse-synapse}=\langle Y_{synapse}^{i}\times Y_{synapse}^{j}\rangle ;\;\;\\ CA_{neuron-glia}=\langle Y_{neuron}^{i}\times Y_{glia}^{j}+Y_{glia}^{i}\times Y_{neuron}^{j}\rangle ;\;CA_{neuron-synapse}=\langle Y_{neuron}^{i}\times Y_{synapse}^{j}+Y_{synapse}^{i}\times Y_{neuron}^{j}\rangle ;\\ CA_{glia-synapse}=\langle Y_{glia}^{i}\times Y_{synapse}^{j}+Y_{synapse}^{i}\times Y_{glia}^{j}\rangle \end{array}$$

For each network, we also introduced internal (intra-network) cellular association indices for six types of associations: neuron–neuron, glia–glia, synapse–synapse, neuron–glia, neuron–synapse, and glia–synapse, wherein each of these indices was calculated as a weighted average of $CA_{i,j}$ (*i* ≠ *j*) in Equation (8) across all combinations of ROIs in the *n*-th network (similar to NCS in Equation (4)). All ROIs in these calculations were weighted by their number of voxels.

### 2.4. Analysis Based on T1w/T2w Approach

To further understand the role that different parts of the neuron play in the formation of brain functional connectivity, we could compare our R2t*-based results with a ratio of T1-weighted (T1w) to T2-weighted (T2w) images as a proxy related to the cortical tissue myelin content proposed by Glasser and Van Essen [29]. While this proxy does not represent a quantitative measure of myelin content, for example, as discussed in [41], it was successfully used for mapping the cytoarchitecture of human cortical areas [42]. By using T1w and T2w data from the same HCP subjects that are used in this paper for the rs-fMRI analysis, we calculated a Myelin Index ($Y_{myelin}^{n};\;n=1,2,\ldots ,17$) as a ratio of T1w to T2w images) for all ROIs in Yeo’s networks. Similar to cellular association indices, we also introduced indices characterizing the myelin associations in each network.

## 3. Results

### 3.1. The Strength of Resting-State Functional Networks Is Significantly Associated with the Neuron–Neuron, Neuron–Glia, and Neuron–Synaptic Structural Circuits in the Human Brain Cortex

**Figure 1:**
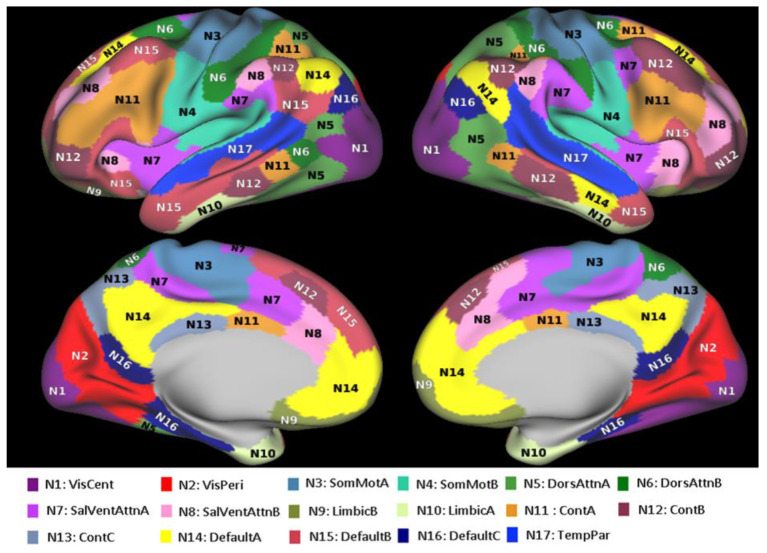
Network parcellation of Yeo’s 17 networks. The 17 networks include the following regions: N1: VisCent–Visual A (22 ROIs); N2: VisPeri–Visual B (18 ROIs); N3: SomMotA–Somatomotor A (30 ROIs); N4: SomMotB–Somatomotor B (21 ROIs); N5: DorsAttnA–Dorsal Attention A (16 ROIs); N6: DorsAttnB–Dorsal Attention B (16 ROIs); N7: SalVentAttnA–Salience/Ventral Attention A (25 ROIs); N8: SalVentAttnB–Salience/Ventral Attention B (14 ROIs); N9: LimbicB–Limbic B (9 ROIs); N10: LimbicA–Limbic A (11 ROIs); N11: ContA–Control A (22 ROIs); N12: ContB–Control B (18 ROIs); N13: ContC–Control C (8 ROIs); N14: DefaultA–Default A (23 ROIs); N15: DefaultB–Default B (25 ROIs); N16: DefaultC–Default C (10 ROIs); and N17: TempPar–Temporal Parietal (12 ROIs). Further details on the networks’ structure can be found in [7,30].

In this paper, we provide a data analysis based on a structure of 300 gwMRF ROIs combined in 17 resting-state networks developed by Yeo et al. [7,30]. These networks are presented in Figure 1. We selected a gwMRF brain parcellation scheme [30] because it exhibits an improved functional connectivity homogeneity compared to other parcellations [42,43,44,45] and provides a sufficient number of parcels (ROIs) to delineate brain cortical anatomical structures as discussed by Van Essen et al. [31].

**Figure A2:**
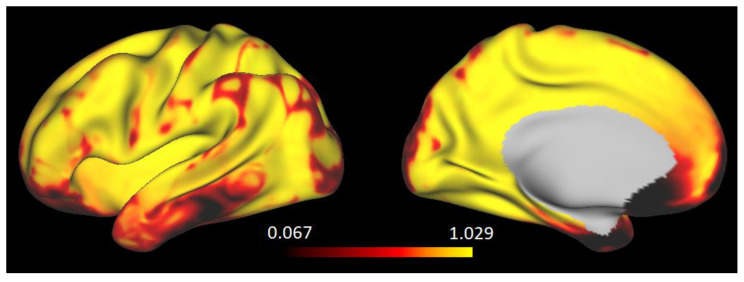
Map characterizing magnetic field inhomogeneities in the human brain. Data obtained from the phase information of the qGRE signal by means of the voxel spread function approach [25]. The yellow color designates areas lightly affected by magnetic field inhomogeneities. The black color designates severely affected areas. The scale bar shows values of the F-function at the gradient echo time TE = 40 ms. The F-function shows a fraction of the signal not affected by the loss due to the field inhomogeneities, ranging from 1 (no signal loss) to 0 (full loss of signal). Images show a significant loss of the signal in both limbic networks, thus adversely affecting the quantitative analysis of both rs-fMRI and qGRE data.

Using qGRE data, we analyzed the contribution of magnetic field inhomogeneities in the signal formation of all 17 networks and found that the two limbic networks are affected the most. In fact, the signals from the networks Limbic A and Limbic B had more than 88% and 80% of voxels significantly affected by the background magnetic field inhomogeneities, respectively, while all other networks had on average only 9% of “bad” voxels (see Appendix A, Figure A2 for details). Consequently, we omitted limbic networks from further consideration.

**Figure 2:**
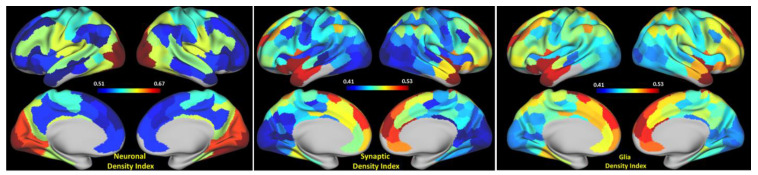
Surface maps of neuronal, glia, and synaptic density indices (qGRE proxy for the neuronal, synaptic, and glia cells’ densities) for 15 networks. Data representing the results in this figure are shown in Appendix A, Table A1.

The theoretical approach described in the Methods section allows—based on Equations (1) and (2)—for the calculation of neuronal, synaptic, and glia cells’ indices (qGRE proxy parameters proportional to the neuronal, synaptic, and glia cells’ densities) for each network. The R2t* maps were obtained from 16 healthy volunteers aged 23 to 35 years as described in the Methods section, projected on 300 selected ROIs and averaged together. Table A1 in Appendix A shows a variation of R2t* measurements across the subjects. Images representing mean neuronal, glia, and synaptic density indices are presented in Figure 2.

**Figure 3:**
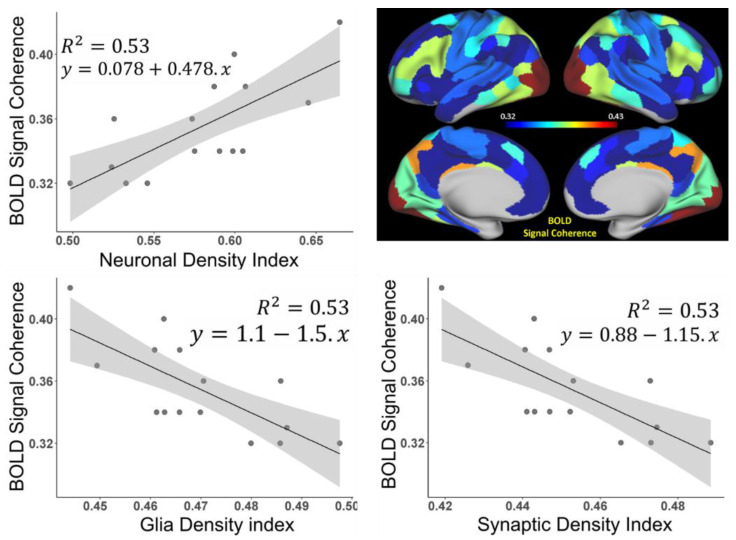
Association between the BOLD signal coherence in 15 networks and neural, glia, and synaptic density indices in these networks (limbic networks are omitted). Neuronal (*Y_neuron_*), glia (*Y_glia_*), and synaptic (*Y_synapse_*) density indices were calculated from R2t* values using Equations (1) and (2) and were then averaged for each network. Shaded areas indicate 95% confidence intervals. The R^2^ values for all three correlations are practically identical because of previously established correlated spatial distributions of neurons, glia, and synapses in the healthy human cortex [27,28], which is reflected by the relationship between glia (*Y_glia_*) and synaptic (*Y_synapse_*) indices to the Neuronal Index (*Y_neuron_*) by means of Equation (2). The data corresponding to all three plots are listed in Table A1.

Plots in Figure 3 represent the correlations between the BOLD signal coherence in 15 networks and neural, glia, and synaptic density indices in these networks. Data show strong positive associations with the mean Neuronal Density Index and negative associations with glia and synaptic density indices. This means that neurons, not glia cells and synapses, are mostly responsible for the coherence of the BOLD signal. The strong negative association of the BOLD signal coherence with glia and synaptic density indices is due to the fact that the densities of glial cells and synapses are negatively associated with neuronal density in the corresponding regions, as presented in Equation (2).

Even though the strength of the BOLD signal coherence in functional networks is mostly associated with the networks’ neuronal content (Figure 3), the functional connectivity of the brain is not solely dependent on the neurons; it also includes glial cells (astrocyte, microglia, and oligodendrocytes) which significantly influence the structural and functional connectivity governed by neurons [16]. Obviously, the factors defining the strength of the functional connectivity between two ROIs are the actual neuronal pathways connecting these ROIs and the cellular composition of these ROIs. Here, we focused on the latter: the ROIs’ cellular composition.

**Figure 4:**
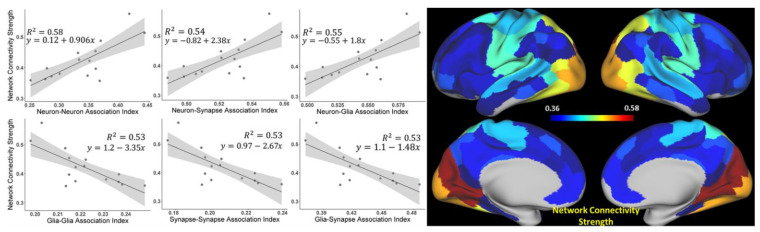
Relationships between network connectivity strength (NCS) and indices characterizing cellular associations between neurons, synapses, and glia cells. Plots show correlations between the resting-state network connectivity strength NCSn and cellular associations strength $CAS^{n}$ (index *^n^* is omitted in the figure) of these networks for six types of cellular associations (neuron–neuron, glia–glia, synapse–synapse, neuron–glia, neuron–synapse, and glia–synapse) for 15-networks. Shaded areas indicate 95% confidence intervals. Images represent surface maps of NCS for 15 networks.

Results in Figure 4 show correlations between the intra-network connectivity strength *NCS_n_,* as expressed in Equation (4), and the cellular association strength $CAS^{n}$, as expressed in Equation (8), of these networks for six types of cellular associations (neuron–neuron, glia–glia, synapse–synapse, neuron–glia, neuron–synapse, and glia–synapse). Data illustrates that the neuron–neuron (*CAS_neuron-neuron_*), neuron–glia (*CAS_neuron-glia_*), and neuron–synapse (*CAS_neuron-synapse_*) associations show strong positive correlations, suggesting that these associations, as dominant features, contribute to the inter-ROIs’ resting-state functional connectivity.

We also tested for associations between the intra-network connectivity strength in 15 networks (see values in Table A1) and the mean values of neural, glia, and synaptic density indices in these networks (also presented in Table A1). The trends are similar to those shown in Figure 3 for BOLD signal coherences with corresponding R^2^ values of 0.56, 0.54, and 0.54.

**Figure A3:**
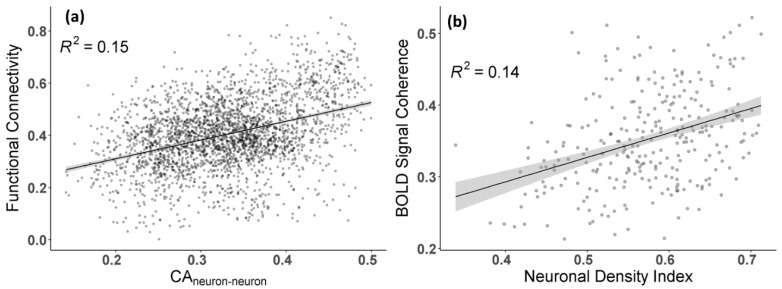
Functional–structural associations. (**a**): Association between the functional connectivity strength as defined based on 280 ROIs and the cellular association between the neurons in these ROIs. The correlation is highly significant with *p* = 9 × 10^−98^. (**b**): Association between the BOLD signal coherence in 280 ROIs and the Neural Density Index in these ROIs. The correlation is highly significant with *p* = 5.3 × 10^−11^. In both curves, the ROIs in the limbic networks are omitted. Shaded areas indicate 95% confidence intervals.

While data in Figure 3 and Figure 4 show network-wide associations between the BOLD signal coherence vs. Neural Density Index and network connectivity strength vs. cellular association strength between cells, respectively, Figure A3 in Appendix A shows similar associations based on 280 individual ROIs (limbic network-associated ROIs were excluded), which are highly significant (*p* = E-11 and E-98 correspondingly).

**Figure A4:**
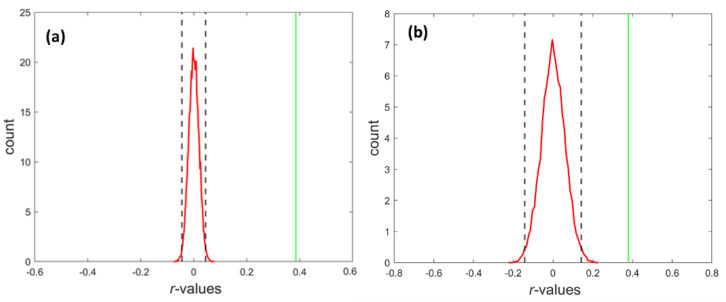
Permutation test analysis (randomization test). (**a**): The functional connectivity vs. structural association between neurons Y (neuron–neuron). The curve shows the distribution of r-values calculated between Y (neuron–neuron) and randomly permuted FC 20,000 times. (**b**): The BOLD signal coherence vs. the Neuronal Density Index (NDI). The curve shows the distribution of r-values calculated between NDI and randomly permuted 10,000 times BOLD signal coherence. In both plots, the 99% percentiles of the distribution lines are marked by black dashed lines, while the r-values without permutation are marked as green lines and are well outside the black lines. The result shown in the figures validates a significant difference in both cases with *p*-values <0.000001.

We also tested for the randomness of our correlation result in Figure 3 (BOLD signal coherence vs. Neuronal Density Index) and Figure 4 (functional connectivity (FC) vs. cellular association strength between neurons) by means of a permutation test (randomization test). The permutation test for the BOLD signal coherence vs. Neuronal Density Index was done by randomly permuting the BOLD signal coherence 10,000 times and calculating the correlation with the Neuronal Density Index for each permutation. The curve in Figure A4b in Appendix A illustrates the distribution of obtained r-values, validating a significant difference with the *p*-value < 0.000001. Similarly, the functional connectivity strengths between 280 ROIs (excluding limbic networks) were randomly permuted 20,000 times and correlated with the cellular association strength between neurons for each permutation. The curve in Figure A4a in Appendix A shows the distribution of obtained r-values, thus validating a significant difference (*p*-value < 0.000001).

**Figure A5:**
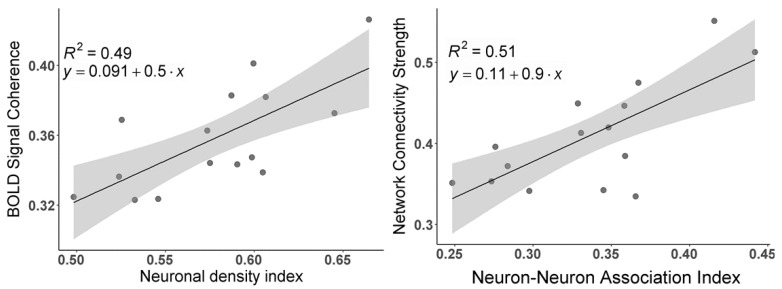
Statistical sufficiency of the sample size. To demonstrate the statistical sufficiency of our sample size (N = 183), we ran an analysis similar to that presented in Figure 3 and Figure 4 of the main text using a smaller sample size (N = 100). Results presented in Figure A5 show practically the same correlations with only slightly smaller R^2^ values.

To demonstrate the statistical sufficiency of our sample size (N = 183), we ran analyses similar to those presented in Figure 3 and Figure 4 using a smaller sample size (N = 100). Results presented in Appendix A, Figure A5 show practically the same correlations with only slightly smaller R^2^ values.

**Figure 5:**
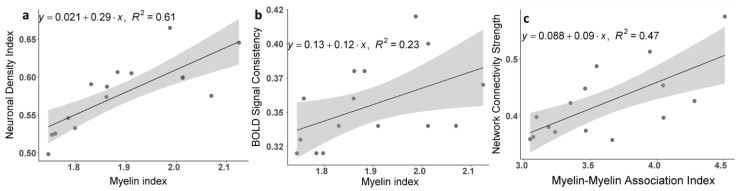
Association between qGRE and T1w/T2w results based on 15 networks. (**a**) Correlation between the Neuronal Density Index and Myelin Index. Points represent average values for individual networks. Shaded areas indicate 95% confidence intervals. The Myelin Index was calculated based on T1w/T2w images. (**b**) Correlations between the strength of the BOLD signal coherence and average Myelin Index in the networks. (**c**) Correlation between the resting-state intra-network connectivity strength, NCS, and Myelin–Myelin Association Index calculated based on the myelin indices in the connected ROIs (similar to that described by Equations (4) and (8)).

Data in Figure 5 show associations between cortical tissue in the Myelin Index calculated based on the ratio of T1w/T2w images and the qGRE-based calculated Neuronal Density Index (Figure 5a), BOLD signal coherence (Figure 5b), and network connectivity strength (Figure 5c).

### 3.2. Brain Cortical Cellular Composition Shows the Strongest Association with the Resting-State BOLD Signal Coherence and Network Connectivity in the Infra-Slow Frequency Range of Neuronal Activity

**Figure 6:**
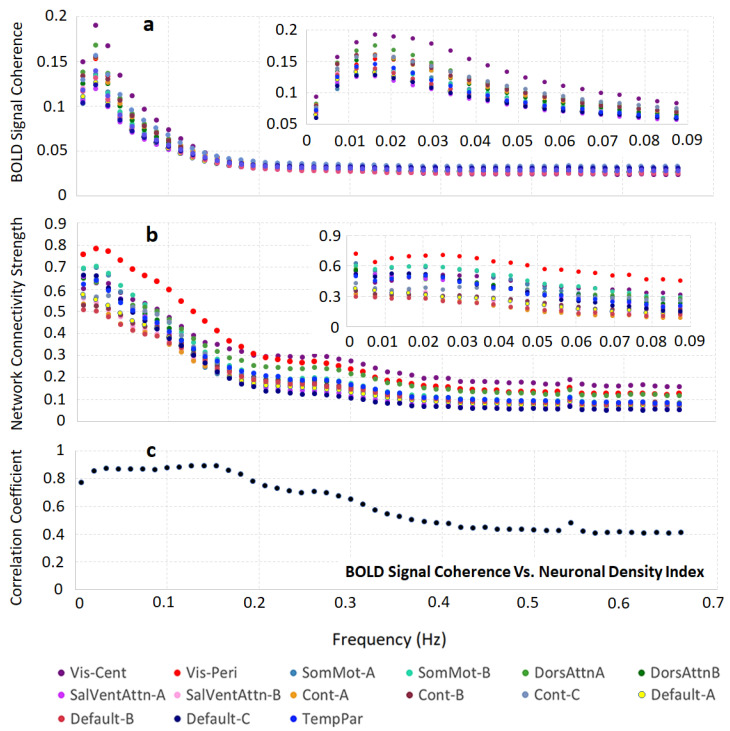
Relationship between the frequency content of the resting-state BOLD signal and the brain cellular structure. (**a**) BOLD signal coherence (mean standard deviations of the rs-fMRI signal) in 15 networks as a function of the rs-fMRI signal frequency content. (**b**) The intra-network connectivity strength (NCS) of the 15 networks as a function of the rs-fMRI signal frequency content. Both panels (**a**,**b**) show relatively sharp peaks for all networks between 0.01 Hz and 0.03 Hz that are further illustrated in the insets. (**c**) Correlation coefficient between the BOLD signal coherences in 15 networks and tissue the neuronal content of these networks (as in Figure 3) as a function of the rs-fMRI signal frequency content. The frequency resolution in the main graphs is 0.014 Hz and in the inset it is 0.0047 Hz. Data show the strongest correlation in a rather broad infra-slow frequency range between 0.01 and 0.16 Hz.

Data in Figure 3 show that the BOLD signal coherence varies significantly among networks and strongly correlates with the networks’ neuronal density. Similar behavior was seen for the network connectivity strength, which also depends on the brain cellular composition, though in a different manner; the intra-network connectivity strength shows the strongest correlation with intra-network neuron–neuron, neuron–synaptic, and neuron–glia associations between networks’ ROIs. These results were obtained by analyzing the time courses of rs-fMRI signals without accounting for the frequency content of this signal. At the same time, only the infra-slow (below 0.1 Hz) frequency fluctuations of the resting-state BOLD signals were usually considered as a signature of the neuronal activity [3,32,33,34,35,36]. To investigate the detailed relationship between cellular composition and frequency components of the resting-state BOLD signal, we converted the time series data into 50 consecutive frequency bands, covering frequency domains 0.01–0.60 Hz (see Methods section), and analyzed different frequency components independently. The results for the 15 networks are presented in Figure 6. Figure 6a shows the BOLD signal coherence (mean standard deviation of the rs-fMRI signal) in each network as a function of the rs-fMRI signal frequency. We found that while different networks exhibit different signal strength coherences, the general trend is quite similar: the strongest coherence of BOLD signal fluctuations was present in the frequencies below 0.16 Hz, with the peak between 0.01 and 0.03 Hz. The NCS calculated in terms of the mean intra-network correlation coefficient for each of the 15 networks as a function of the rs-fMRI signal frequency content can be seen in Figure 6b, which also displays increased connectivity strengths in a similar frequency range below 0.16 Hz, with the peak between 0.01 and 0.03 Hz. Figure 6c shows the correlation between the BOLD signal coherences in 15 networks and the tissue neuronal content of these networks (as in Figure 3) as a function of the rs-fMRI signal frequency. Interestingly, the strongest correlation exists not only in the frequency range where the BOLD signal coherence and NCS are the strongest, but in a rather broad infra-slow frequency range between 0.01 and 0.16 Hz.

## 4. Discussion

While numerous specific mechanisms involved in neuronal signal transmissions between different parts of the CNS are well understood, understanding the interrelationships between these mechanisms and how they contribute to brain function is still an elusive goal of neuroscience. A great number of papers are now devoted to one branch of this goal: studying brain functional networks reflecting intrinsic brain activity in the resting state (e.g., see [46] and references therein). While significant progress has been achieved in studying resting-state functional networks in health and disease, many questions related to the relationship between resting-state functional organization and underlying brain cellular organization are still not well understood.

Substantial advances in this direction have been achieved by utilizing the information on brain genetic organization available from the Allen Human Brain Atlas (https://portal.brain-map.org/, last accessed 10 October 2021). Richiardi et al. [47] found that functional networks are underpinned by the networks of genes coding for ion channels and synaptic functions. Hawrylycz et al. [48] demonstrated that genes in the neuron-associated networks showed higher preservation between human brains and were related to functionally relevant circuitry. Furthermore, Goyal et al. [49] were able to differentiate between the use of aerobic glycolysis in networks, which was associated with the presence of genes related to synapse formation and growth (i.e., transcriptional neotony).

By using information on the spatial distribution of gene expression profiles in the human brain, as provided by the Allen Human Brain Atlas, Wen et al. [26] identified three gene structural networks related to brain neuronal, glia, and synaptic structures. Importantly, a strong association between these networks and the R2t* metric of the qGRE MRI signal [22] was established in [26]. This showed that the in vivo measurement of the major baseline tissue cellular-specific component of qGRE signal decay rate parameter R2t* (t stands for tissue) provides a unique genetic perspective into the cellular constituents of the human cortex and can serve as a previously unidentified link between the cortical tissue cellular composition and MRI signal. The analysis of the genetically derived brain cellular composition [26] was in good agreement with direct histological measurements by Herculano-Houzel [27] and other previous findings [29,50,51].

It is worth noting that the method used in [26] to establish a relationship between the R2t* metric and brain tissue neuronal density is not as different from traditional histology as one might think. Indeed, in traditional histology, an MRI metric (R2t* in our case) would be correlated with the count of histological markers specific to mature neurons, to other markers specific to other cells such as astrocytes, and to structural components such as myelin. In [26], instead of using histological markers, published gene expression profiles (Microarray Data: Allen Brain Atlas: Human Brain (brain-map.org, last accessed 10 October 2021)) were used with their relationship to neurons established using available tools such as the ToppGene portal (https:/toppgene.cchmc.org, last accessed 10 October 2021), which includes 19,061 genes in the “Cellular Component” category and 23,956 genes in the “Coexpression Atlas” category. The sensitivity of the gene enrichment analysis was demonstrated using DAVID Bioinformatics Resources (https://david.ncifcrf.gov/, last accessed 10 October 2021). Results showed that gene expression profiles affiliated with neuronal cellular and subcellular compartments correlated strongly with R2t*.

In this paper, we used quantitative relationships established in [26] between the R2t* metric of the quantitative Gradient-Recalled Echo (qGRE) MRI signal and the human brain cellular composition to study in vivo interrelationships between human brain resting-state functional connectivity, which is known to provide insight into large-scale brain circuit organization [2,3] and underlying brain cellular organization.

Our functional connectivity analysis is based on the resting-state data obtained from the HCP1200 dataset (February 2017 release) of young adults aged between 22 and 35 years [5]. To analyze resting-state functional connectivity data, we used brain parcellation into individual functional units and the brain resting-state networks proposed in [7,30] that allow for a significant reduction of large volumes of resting-state functional connectivity data, but also provide a sufficient number of parcels (ROIs) to delineate the importance of brain cortical anatomical structures, thus satisfying the important features of fMRI data analysis discussed by Van Essen et al. [31].

Our results elucidate the relationship between the components of the human brain cellular composition (neurons, glia cells, and synapses) and the BOLD signal characteristics of the resting-state functional networks. In our approach, the functional connectivity inside the individual functional units (ROIs) is characterized by the coherence of the BOLD signals from the voxels/vertices comprising functional units (Equation (3)), while the intra-network functional connections are characterized by the coherence of the global BOLD signals generated by the functional units comprising these networks (Equation (4)). Our analysis revealed the relationships between brain functional properties and the cellular content. The general trend indicates that the functional units, with higher concentration of neurons and correspondingly lower concentrations of glial cells and synapses, display stronger coherence of the rs-fMRI BOLD signals in the individual functional units (Figure 3 and Figure A3 in Appendix A). This result reveals that the synchrony of the connections between brain cellular circuits in individual functional units is mostly associated with the neuronal content; a higher concentration of neurons leads to stronger functional connections. To establish a joint impact of the cellular content of functional units on the between-unit resting-state functional connectivity strength, we considered six cellular associations defined by the components of the functional units’ cellular composition, such as neuron–neuron, glia–glia, synapse–synapse, neuron–glia, neuron–synapse, and glia–synapse (Equation (8)). The correlations between these associations and the functional connectivity strength suggest that the synchrony of connections between cellular circuits belonging to different functional units in the network is associated not only with the neuron–neuron associations between functional units but also with the neuron–synapse and neuron–glia associations (Figure 4).

By comparing Figure 3 and Figure 4 with Figure A3 in Appendix A, we conclude that the associations between the cellular compositions of functional units and the synchrony of their cellular circuits (defined by both BOLD signal coherence and functional connectivity Strength) are stronger for functional units belonging to the same networks (Figure 3 and Figure 4) as compared with the general whole-brain trends presented in Figure A3 in Appendix A. The R^2^ values in Figure 3 and Figure 4 are greater or equal to 0.53, which is more than three times greater than the R^2^ values in Figure A3 (R^2^ = 0.14 and 0.15), thus the regression analysis represented in Figure 3 and Figure 4 explains the three-times more variation in the data than the regression analysis represented in Figure A3. These results suggest that (among other functions) the cellular organization of the human cortex provides significant support to the brain functional connectivity by synchronizing the intra-networks cellular circuits.

The strong role of the neuronal–glia association in forming strong network connections is also in agreement with the role that glial cells play by providing metabolic [52,53] and regulatory [54] support for neurons, as well as support for neurite outgrowth and neuronal guidance [17,18]. Moreover, neuron–glia cross-talk leads to synaptic formation and remodeling. In absence of glia, neurons generate weak synapses [17,21]. The dependence of the intra-network connectivity strength on the neuron–synapse relationship could also have been expected because they operate as a unit in conducting brain electric currents.

To further clarify the role that different parts of the neuron play in the formation of brain functional connectivity, we can compare our R2t*-based results with a T1w/T2w-derived proxy related to the cortical tissue myelin content proposed [29] and successfully used [42] for mapping human cortical areas by Glasser and Van Essen. An association between T1w/T2w myelin proxy and gene profiles from an Allen Human Brain Atlas was also established in [55]. Data in Figure 5a show a very strong (R^2^ = 0.61) correlation between measurements of the Neuronal Density Index (NDI) and the T1w/T2w-derived Myelin Index (MI) across the 15 networks (limbic networks were omitted from this consideration for the same reasons that R2t* and functional connectivity data were omitted). This result is consistent with previous findings that the neuronal densities tend to be higher in areas of high myelin content and low in areas of low myelin content [29,50] that have more complex intracortical circuitry (i.e., larger dendritic field sizes and larger dendritic arbors) [51]. At the same time, data suggest that not all parts of the R2t*-based calculated NDI are associated with the presence of myelinated neuronal processes. These results are expected because myelin covers only part of the neuron, i.e., myelinated axons, while R2t*-based NDI comprises all parts of the neuronal structure, i.e., cell body, myelinated and non-myelinated axons, and dendrites.

The conclusion that the qGRE-derived NDI also has additional contributions of myelinated axons from remaining neuronal components—dendrites and soma—is further supported by data in Figure 5b,c. Indeed, data in Figure 5b show that the correlation between the BOLD signal coherence and Myelin Index is much weaker (R^2^ = 0.23) than the correlation between the BOLD signal coherence and NDI (R^2^ = 0.51, Figure 5c). Subsequently, a comparison of data in Figure 4 and Figure 5c shows that the intra-network connectivity strength has a stronger association with neuron–neuron associations (R^2^ = 0.53] than with the associations due to the myelinated processes (R^2^ = 0.47). This might suggest that the qGRE NDI metric has a stronger association with the brain cellular “functionality” than the T1w/T2w-derived Myelin Index. These results are quite instructive. They suggest that the BOLD signal coherence in the individual brain ROIs comprising functional connectivity networks might be mostly associated with the non-myelinated parts of the neurons, namely dendrites and soma. At the same time, the inter-ROIs’ connectivity has a significantly stronger contribution from myelinated axons needed to secure inter-unit within-network functional connectivity.

In this paper, we have also provided a detailed analysis of the “frequency content” of the resting-state BOLD signal and a corresponding strength of the resting-state functional connectivity in relation to the underlying cellular composition. It is well appreciated that the resting-state BOLD signal originates from a specific element of brain activity, i.e., infra-slow activity with frequencies approximately below 0.1 Hz, which is thought to be linked in this interval to neuronal activities [3,32,33,34,35,36]. Our data are in agreement with this notion: for all networks, the resting-state BOLD signal coherence and intra-network connectivity strength are not monotonic functions of the frequency, with rather sharp peaks in the infra-slow frequency range between 0.01 and 0.16 Hz (Figure 6a,b). A relationship of these infra-slow BOLD signal fluctuations to neuronal activity is clearly supported by a plot in Figure 6c showing a high correlation plateau in the frequency range of 0.01 to 0.16 Hz on the curve, displaying a correlation between the Neuronal Density Index and BOLD signal coherence.

All these mechanisms lead to a rather broad distribution of resting-state functional networks’ properties. We found that the visual networks (N1 and N2 in Figure 1) express the highest neuronal density, although with the lowest density of glial cells and synapses, and the strongest intra-functional-unit BOLD signal coherence and intra-network connectivity. The DMN (N14, N15, and N16 in Figure 1) shows relatively low intra-functional-unit BOLD signal coherence and intra-network connectivity strength, but a significant diversity of cellular constituents. The DMN part B exhibits a remarkably balanced cellular content; the Neuronal Density Index (0.51) was almost exactly equal to the glia density (0.49) and synaptic density (0.48) indices. At the same time, the other parts of DMN (A and C) that include temporal and inferior parietal lobules as well as the dorsal prefrontal, precuneus posterior cingulate, medial prefrontal, retrosplenial, and parahippocampal cortices have more condensed neuronal content with relatively lower concentrations of synapses and glia cells. This network is affiliated with temporal, inferior parietal, dorsal prefrontal, lateral prefrontal, and ventral prefrontal brain regions. These results can potentially be helpful in understanding the unique features of DMN and its very prominent role in the overall organization of the brain [56], in addition to its targeting of Alzheimer disease [57].

As the study of the resting-state functional connectivity within and among the large networks of the human brain has moved forward, the concept of hierarchies among these networks has emerged as a major area of interest (for example, see [35,58] and the references therein). Broadly speaking, networks are positioned according to a variety of features beginning with the sensorimotor cortices and ascending to association cortices, with the brain’s default mode network at the top of this hierarchy. Networks at the top of the hierarchy are less myelinated [59], exhibit higher levels of aerobic glycolysis [59,60], and harbor neotenous genes [49]. Together these features relate to the presence of synapses and their role in both growth and plasticity. Our data would add that networks at the top of the hierarchy have enriched synaptic structures (Figure 2). This may be relevant to our understanding of the functional feature of networks at the top of the hierarchy as they integrate incoming information more slowly than, for example, primary sensory areas [35,61].

## 5. Conclusions

In this paper, we provided a detailed analysis of the associations between the human brain cellular composition and functional networks identified by the resting-state BOLD signal. Our results show that the brain regions with higher concentrations of neurons, but relatively lower concentrations of glial cells and synapses, support strong connections between cellular circuits in the network-defined functional units, leading to a strong BOLD signal coherence in individual functional units. Furthermore, our results show that a significant contribution to between-unit connectivity is provided by the neuron–neuron, neuron–synaptic, and neuron–glia associations between cellular circuits.

These relationships lead to a rather broad distribution of the resting-state functional networks’ properties. We found that visual networks have the strongest BOLD signal coherence with the highest neuronal density (but lowest density of glial cells and synapses) as well as the strongest intra-network connectivity. The Default Mode Network (DMN) shows relatively low BOLD signal coherence and network connectivity strength but also a significant diversity of cellular circuits, reflecting the DMN’s prominent role in the overall organization of the brain and hierarchy of functional networks.

## Data Availability

Data generated during this study are fully described in the paper. Additional information can be obtained from the corresponding author upon request.

## Appendix A

**Table A1:** Summary of *R2t** and the cellular densities in the 15 networks. Columns 2 and 3 represent the mean and standard deviation of the *R2t** distribution across 16 subjects (values are in 1/sec). Columns 4–6 represent the mean values of the neuronal, glia, and synaptic density indices (qGRE proxy for the neuronal (NDI), synaptic (SDI), and glia (GDI) cells’ densities). Data in columns 4–6 correspond to the maps in Figure 2 of the main text. Data in columns 7 and 8 represent the mean values of the BOLD signal coherence and functional connectivity strength (FCS) corresponding to the data in Figure 3 and Figure 4 of the main text.

NETWORKS	*R2t**Mean	*R2t**STD	NDI	GDI	SDI	BOLD Coherence	FCS
**Vis-Cent**	19.67	0.8	0.66	0.44	0.42	0.51	0.42
**Vis-Peri**	18.86	0.48	0.65	0.45	0.43	0.57	0.37
**SomMot-A**	16.95	0.82	0.58	0.47	0.45	0.43	0.34
**SomMot-B**	18.02	0.78	0.6	0.46	0.44	0.45	0.34
**DorsAttnA**	17.88	0.67	0.61	0.46	0.44	0.49	0.38
**DorsAttnB**	17.13	0.79	0.57	0.47	0.45	0.45	0.36
**SalVentAttn-A**	16.61	0.68	0.53	0.49	0.47	0.37	0.32
**SalVentAttn-B**	16.34	0.71	0.52	0.49	0.47	0.36	0.33
**Cont-A**	17.69	0.73	0.59	0.47	0.45	0.37	0.38
**Cont-B**	16.55	0.74	0.53	0.49	0.47	0.40	0.36
**Cont-C**	17.87	0.96	0.6	0.46	0.44	0.40	0.40
**Default-A**	16.6	0.71	0.55	0.48	0.47	0.38	0.32
**Default-B**	16.29	0.83	0.5	0.5	0.49	0.36	0.32
**Default-C**	18.06	0.71	0.6	0.49	0.44	0.36	0.34
**TempPar**	17.81	0.68	0.59	0.47	0.45	0.42	0.34
